# Keystone Veterinary Medical Association

**Published:** 1895-04

**Authors:** W. L. Rhoads

**Affiliations:** Secretary


Secretary.

### KEYSTONE VETERINARY MEDICAL ASSOCIATION.

THE March meeting of the Keystone Veterinary Medical Association was called to order by President Lintz at the office of Dr. W. H. Hoskins, No. 3452 Ludlow Street, on the 12th ult.

The following members were present: Drs. Bridge, Eves, Goentner, Hoskins, J. R. Hart, Lintz, Rhoads, and McAnulty ; Dr. Allen being the only visitor present.

Dr. Hoskins, as Chairman of the Legislative Committee, reported the bill to place a veterinarian officially on the State Board of Agriculture to have passed both houses, and only wants the Governor's signature to become a law.

The bill to establish a State Board of Veterinary Examiners is in the same position it was a month ago, and now stands a poor show of being considered this session.

The Veterinarians' Examining Board of Ohio meets in April, and have invited Dr. Hoskins to be present.

After this report the qualifications of the known applicants for State Veterinarian under the new law were thoroughly considered and discussed. It was urged that at this particular time a man should be selected who is particularly well fitted and naturally adapted for this work, that the people might learn to repose greater confidence in the profession.

On motion, the President appointed Drs. Hoskins, Bridge, and J. R. Hart a Committee to select from the applicants the one they thought best fitted to honor and advance the profession as State Veterinarian. The Committee after due deliberation selected Dr. Leonard S. Pearson as best qualified and adapted to this work.

The Board of Censors were instructed to look for a larger meeting-room, as the attendance made more room essential. They were also instructed to consider the advisability of reducing the annual dues and see to a revision and reprinting of the By-laws.

The Board of Censors now reported favorable consideration of Dr. Leonard S. Pearson's application for membership, and he was unanimously elected.

Dr. Goentner reported a very interesting case of pernicious anæmia in a running stallion.

The reading of papers was postponed, as the time was taken up with discussion.

The meeting adjourned to meet at the home of Dr. Kooker April 9th.

W. L. RHOADS,
Secretary.

